# Socioeconomic variation in characteristics, outcomes, and healthcare utilization of COVID-19 patients in New York City

**DOI:** 10.1371/journal.pone.0255171

**Published:** 2021-07-29

**Authors:** Yongkang Zhang, Dhruv Khullar, Fei Wang, Peter Steel, Yiyuan Wu, Duncan Orlander, Mark Weiner, Rainu Kaushal

**Affiliations:** 1 Department of Population Health Sciences, Weill Cornell Medical College, New York, NY, United States of America; 2 Department of Medicine, Weill Cornell Medical College, New York, NY, United States of America; 3 NewYork-Presbyterian Hospital, New York, NY, United States of America; 4 Department of Emergency Medicine, Weill Cornell Medicine, New York, NY, United States of America; 5 Department of Pediatrics, Weill Cornell Medical College, New York, NY, United States of America; Azienda Ospedaliero Universitaria Careggi, ITALY

## Abstract

**Objectives:**

There is limited evidence on how clinical outcomes differ by socioeconomic conditions among patients with coronavirus disease 2019 (COVID-19). Most studies focused on COVID-19 patients from a single hospital. Results based on patients from multiple health systems have not been reported. The objective of this study is to examine variation in patient characteristics, outcomes, and healthcare utilization by neighborhood social conditions among COVID-19 patients.

**Methods:**

We extracted electronic health record data for 23,300 community dwelling COVID-19 patients in New York City between March 1^st^ and June 11^th^, 2020 from all care settings, including hospitalized patients, patients who presented to the emergency department without hospitalization, and patients with ambulatory visits only. Zip Code Tabulation Area—level social conditions were measured by the Social Deprivation Index (SDI). Using logistic regressions and Cox proportional-hazards models, we examined the association between SDI quintiles and hospitalization and death, controlling for race, ethnicity, and other patient characteristics.

**Results:**

Among 23,300 community dwelling COVID-19 patients, 60.7% were from neighborhoods with disadvantaged social conditions (top SDI quintile), although these neighborhoods only account for 34% of overall population. Compared to socially advantaged patients (bottom SDI quintile), socially disadvantaged patients (top SDI quintile) were older (median age 55 vs. 53, P<0.001), more likely to be black (23.1% vs. 6.4%, P<0.001) or Hispanic (25.4% vs. 8.5%, P<0.001), and more likely to have chronic conditions (e.g., diabetes: 21.9% vs. 10.5%, P<0.001). Logistic and Cox regressions showed that patients with disadvantaged social conditions had higher risk for hospitalization (odds ratio: 1.68; 95% confidence interval [CI]: [1.46, 1.94]; P<0.001) and mortality (hazard ratio: 1.91; 95% CI: [1.35, 2.70]; P<0.001), adjusting for other patient characteristics.

**Conclusion:**

Substantial socioeconomic disparities in health outcomes exist among COVID-19 patients in NYC. Disadvantaged neighborhood social conditions were associated with higher risk for hospitalization, severity of disease, and death.

## Introduction

The COVID-19 pandemic is an unprecedented public health crisis globally, including in the United States, where New York City (NYC) became the initial epicenter in March 2020 [1–4]. As of early June of 2021, NYC reported approximately 800,000 confirmed cases, over 100,000 hospitalizations, and over 33,000 confirmed deaths [5]. Better understanding the clinical characteristics, outcomes, and patterns of healthcare utilization for COVID-19 patients is important to inform clinical decision-making and public health policy in the current pandemic, including for the growing number of individuals with long-term sequelae of the disease, and for future public-health crises.

Available data on COVID-19 patients in NYC and from other regions are limited in several ways. Most studies have focused on inpatients from a single hospital or a single health system [2–4, 6, 7]. However, many patients with COVID-19 are not hospitalized, and instead receive emergency department care without following hospitalization or are treated in ambulatory settings only. There is also significant variation in clinical characteristics and outcomes across health systems and findings from a single health system may not be generalizable. Most studies have not followed patients after hospital discharge, as data on post-discharge outcomes and utilization are often unavailable [3, 4, 6, 7]. Finally, although some studies have examined racial and ethnic disparities in COVID-19 outcomes [8–17], there is less data on how disadvantaged social conditions are associated with COVID-19 outcomes.

Previous literature has demonstrated that racial and ethnicity disparities are distinct from socioeconomic disadvantages [18–20]. Although patients from racial and ethnic minority groups are more likely to have vulnerable social conditions, socially disadvantaged patients represent a range of racial and ethnic groups. These patients are more likely to have chronic conditions, limited access to healthcare, and other risk factors for adverse outcomes related to COVID-19 [21–23]. Better understanding the independent association between socioeconomic characteristics and COVID-19 outcomes may improve medical care and health outcomes for socially disadvantaged patients.

In this study, we compared patient clinical characteristics, health outcomes, and healthcare utilization by neighborhood social conditions for 23,300 COVID-19 patients in NYC between March 1^st^ and June 11^th^, in the ambulatory, emergency department, and inpatient settings. Using multivariable regressions, we examined the associations of neighborhood social conditions with hospitalization and mortality, adjusting for race, ethnicity, and other patient characteristics.

## Methods

### Study setting and data

For this retrospective cohort study, we obtained data for COVID-19 patients from INSIGHT—a clinical research network funded by the Patient-Centered Outcomes Research Institute that aggregates clinical data from health systems to support clinical research [24, 25]. Health systems affiliated with INSIGHT include NewYork-Presbyterian East (Weill Cornell), NewYork-Presbyterian West (Columbia), Mount Sinai Health System, Montefiore Medical Center, and NYU Langone Medical Center. We linked clinical data with social data at zip-code tabulation area (ZCTA) level from the Robert Graham Center for Policy Studies in Family Medicine and Primary Care [26] and the 2018 American Community Survey [27].

### Study cohort

The INSIGHT COVID-19 database includes all patients who were tested for the SARS-Cov-2 virus infection and treated in the five health systems between March 1^st^ and June 11^th^, 2020. We identified all patients with confirmed COVID-19, defined as having at least one positive laboratory test result on real-time reverse transcription polymerase chain reaction (RT-PCR) or at least one ICD-10 diagnosis code for COVID-19 (some patients may have been tested outside the health systems in this study). For patients with confirmed COVID-19, we identified all COVID-19-related clinical encounters and categorized them into three mutually exclusive groups: (1) patients who were admitted to hospital; (2) patients who presented to the emergency department (ED) but were not hospitalized; (3) patients who had ambulatory visits without any ED visits or hospitalizations.

### Social conditions

We examined patient neighborhood social conditions at the ZCTA level. We first used the Social Deprivation Index (SDI) to measure the overall neighborhood social conditions. SDI is a composite score based on seven socioeconomic characteristics. Although other similar social indices exist, such as Area Deprivation Index or Social Vulnerability Index, we chose SDI as it is publicly available at the ZCTA level [26, 28]. Previous studies have found that SDI is associated with increased risk of poor health outcomes [28, 29].

We also examined five measures that reflect important socioeconomic aspects of a neighborhood, including median household income, percent of residents without a high school degree, percent of residents who are essential workers [30], percent of households with crowding housing conditions (more than one person per room), and unemployment rate.

### Overall patient characteristics and outcomes

For all patients with confirmed COVID-19, we examined demographics and baseline comorbidities. Demographics included age, sex, race (White, Black, Asian, other or unknown), and ethnicity (Hispanic, non-Hispanic, or unknown). Baseline comorbidities included hypertension, diabetes, coronary artery disease, heart failure, chronic obstructive pulmonary disease, asthma, cancer, obesity, and hyperlipidemia. We identified these conditions using established diagnosis codes [31]. We also reported most recent Body Mass Index (BMI) as it is a significant risk factor for poor outcomes of COVID-19. Primary outcomes included hospitalization and mortality, including both inpatient deaths and deaths after hospital discharge recorded in the electronic health record.

### Inpatient characteristics and treatment

For hospitalized patients, we examined locations prior to admission, discharge status (discharge alive or died in hospital), presenting laboratory test results after admission (usually drawn within 24 hours of ED or hospital admission), and length of stay. We also examined treatment and procedures during the hospitalization, including intensive care unit (ICU) admission, mechanical ventilation, renal replacement therapy, and prescriptions of vasopressor agents, steroids, or hydroxychloroquine. Finally, we examined the discharge destinations for those discharged alive.

### Healthcare utilization

For all patients, we examined the setting of the first encounter at which they tested positive or had a COVID-19 diagnosis (ED or ambulatory visit). For hospitalized patients who were discharged alive, we examined healthcare utilization 30-day after discharge. For patients who presented to ED without hospitalization and patients who had only ambulatory visits, we examined healthcare utilization 30-day after the COVID-19 ED visit or ambulatory visit. Healthcare utilization included hospitalization, ED visits, and ambulatory visits.

### Statistical analysis

Our primary analysis focuses on community dwelling patients as social conditions of their residential neighborhoods are more likely to have a direct impact on their health outcomes. For patients who live in long-term care facilities, neighborhood social conditions may be less influential for their health outcome than the environment of the facility. Therefore, we excluded these patients in the primary analysis.

We first examined the geographic distribution of patients based on their residential zip codes. We mapped patient zip codes onto ZCTAs. We categorized all ZCTAs into quintiles based on SDI score. Areas in higher quintiles have more disadvantaged social conditions. We presented overall patient characteristics, inpatient characteristics and treatment, and healthcare utilization by SDI quintiles and compared them between socially disadvantaged patients (SDI quintile 5) and socially advantaged patients (SDI quintile 1). We summarized continuous variables as medians and interquartile ranges (IQRs) and categorical variables as percentages. Missing data were not imputed. For measures with missing values, we reported the effective sample size. All comparisons were made using the Wilcoxon rank sum test for continuous variables and the χ^2^ test for categorical variables.

We examined the association of SDI quintiles with hospitalization among all patients using logistic regressions and with mortality among hospitalized patients using Cox proportional-hazards models. The Cox models for mortality used the time from hospital admission to death as the outcome. Patients who were still alive at the end of the follow-up period were counted as censored. We first fit the logistic regressions and Cox models with indicators of SDI quintiles only. We then adjusted for patient demographics and baseline comorbidities. For the Cox models, we additionally adjusted for presenting laboratory test results. All controls were selected based on clinical relevance, prior literature, and data availability [3, 4, 6, 7, 17]. The adjusted associations indicate the direct relationships between social conditions and COVID-19 outcomes, independent of race, ethnicity, and other patient characteristics. To correct for multiple comparisons, the false discovery rate (q value) was calculated [32]. Q-value < 0.05 was considered statistically significant (i.e., controlling the false discovery rate at 5%) [33, 34].

### Secondary and sensitivity analyses

Our secondary analysis focused on patients who lived in long-term care facilities. As a sensitivity analysis, we examined the association of each of the five social condition measures (e.g., income, education, occupation, housing conditions, and unemployment) with hospitalization and mortality.

All statistical analyses were conducted with STATA 14.0 and R version 3.6.3. We also used the shapefile of the ZCTAs from the US Census Bureau to create a map for the geographical distribution of COVID-19 patients in our sample [35]. The Institutional Review Board of the Weill Cornell Medicine approved this study.

## Results

### Geographical distribution of patients

**Fig 1:**
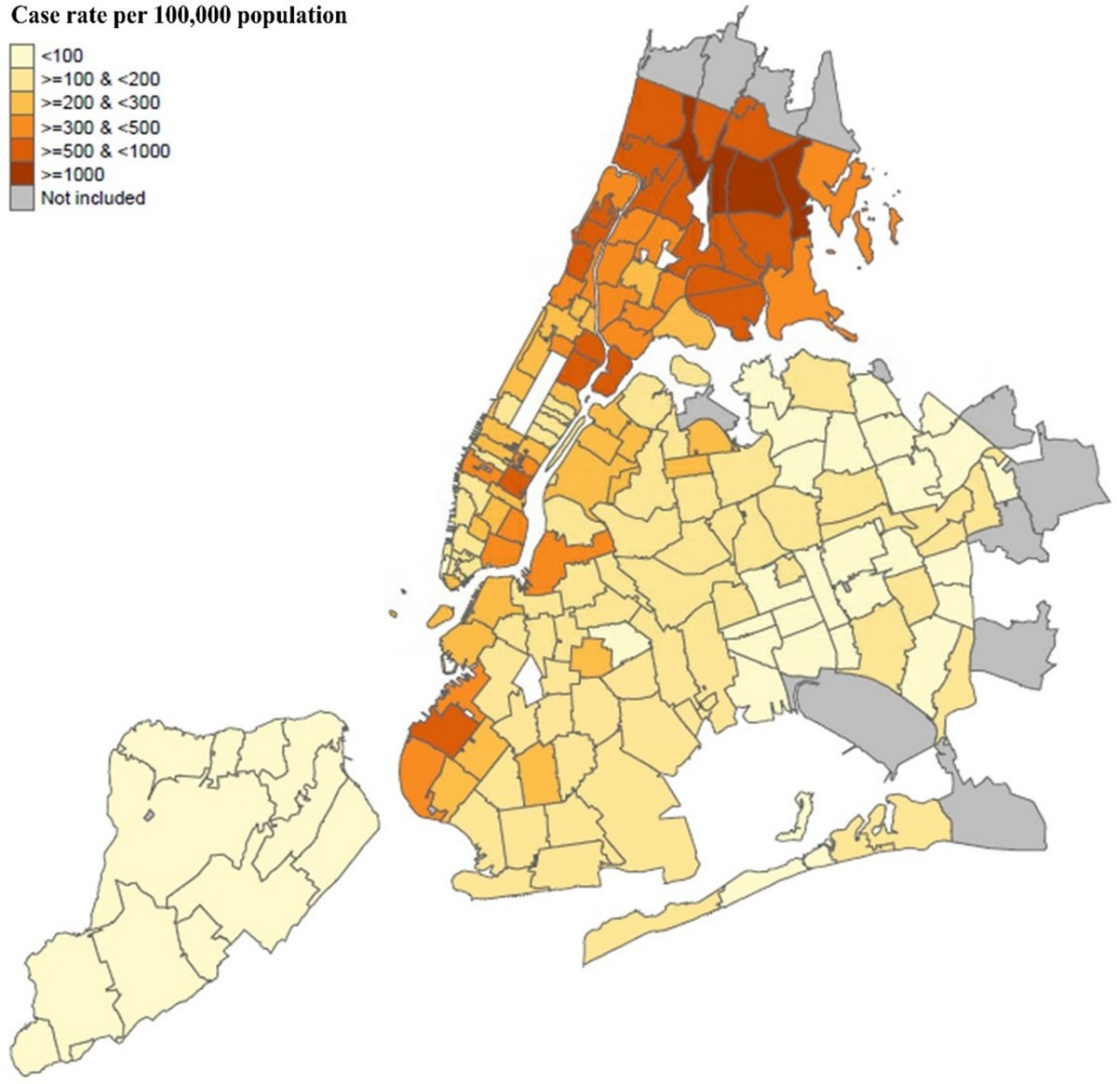
Catchment areas and COVID-19 care rate per 100,000 population in New York City between March 1st and June 11th, by zip code tabulation area. Notes: this map presented distribution of COVID-19 patients with a zip code within the five boroughs of NYC. Patients with a zip code outside five boroughs were not presented in this map.

We identified 23,300 community dwelling patients with COVID-19 from five NYC health systems between March 1^st^ and June 11^th^, 2020. Among these patients, 77.3% (N = 18,009) were from the five boroughs of NYC and the rest were from other parts of New York State or other parts of the New York metropolitan area. Fig 1 presents the distribution of patients by ZCTA in NYC. Areas in the Bronx, Brooklyn, and parts of Queens have a higher COVID-19 infection rate as compared to other areas.

### Socioeconomic variation in overall patient characteristics

Patients with COVID-19 were disproportionately from neighborhoods with disadvantaged social conditions (Table 1). Among all 23,300 COVID-19 patients, 14,135 (60.7%) were from socially disadvantaged areas (SDI quintile 5), although these areas only accounted for 34% of overall population. Only 1,164 (5.0%) were from socially advantaged areas (SDI quintile 1), which accounted for 12% of overall population.

**Table 1:** Overall patient characteristics by quintiles of social deprivation index.

Characteristics	Overall N = 23,300	Social Deprivation Index Quintiles					P value^a^
		Quintile 1 (socially advantaged) N = 1,164	Quintile 2 N = 1,105	Quintile 3 N = 2,613	Quintile 4 N = 4,283	Quintile 5 (socially disadvantaged) N = 14,135	
**Treatment settings, No. (%)**							
**Admitted to ambulatory clinics only**	10,226 (43.9)	764 (65.6)	614 (55.6)	1,494 (57.3)	2,141 (50.0)	5,210 (36.9)	<0.001*
**Admitted to ED only**	3,938 (16.9)	81 (7.0)	103 (9.3)	279 (10.7)	465 (10.9)	3,010 (21.3)	<0.001*
**Hospitalized**	9,136 (39.2)	319 (27.4)	388 (35.1)	837 (32.0)	1,677 (39.2)	5,915 (41.9)	<0.001*
**Age, median (IQR)**	54 (38–68)	53 (39–65)	53 (39–66)	51 (35–66)	53 (37–68)	55 (39–69)	0.002*
**Gender, No. (%)**							
**Female**	11,962 (51.3)	650 (55.8)	586 (53.0)	1,365 (52.2)	2,101 (49.1)	7,260 (51.4)	0.003*
**Male**	11,332 (48.6)	514 (44.2)	519 (47.0)	1,248 (47.8)	2,178 (50.9)	6,873 (48.6)	0.003*
**Other/Unknown**	6 (0.0)	0 (0.0)	0 (0.0)	0 (0.0)	4 (0.1)	2 (0.0)	0.69
**Race, No. (%)**							
**White**	7,319 (31.4)	618 (53.1)	541 (49.0)	1,210 (46.3)	1,771 (41.4)	3,179 (22.5)	<0.001*
**Black**	4,402 (18.9)	74 (6.4)	105 (9.5)	285 (10.9)	673 (15.7)	3,265 (23.1)	<0.001*
**Asian**	1,207 (5.2)	75 (6.4)	88 (8.0)	186 (7.1)	272 (6.4)	586 (4.2)	<0.001*
**Other/unknown**	10,372 (44.5)	397 (34.1)	371 (33.6)	932 (35.7)	1,567 (36.6)	7,105 (50.3)	<0.001*
**Ethnicity, No. (%)**							
**Hispanic**	4,620 (19.8)	99 (8.5)	113 (10.2)	282 (10.8)	534 (12.5)	3,592 (25.4)	<0.001*
**Non-Hispanic**	12,234 (52.5)	724 (62.2)	691 (62.5)	1,575 (60.3)	2,696 (63.0)	6,548 (46.3)	<0.001*
**Unknown**	6,446 (27.7)	341 (29.3)	301 (27.2)	756 (28.9)	1,053 (24.6)	3,995 (28.3)	0.45
**BMI, median (IQR)**	28.3 (24.4–33.7)	27.2 (23.6–31.7)	27.4 (23.8–31.6)	26.8 (23.2–31.0)	27.6 (24.0–32.4)	28.9 (24.8–34.7)	<0.001*
**BMI level, No. (%)**							
**<18.5 (%)**	456 (2.0)	12 (1.0)	22 (2.0)	44 (1.7)	85 (2.0)	293 (2.1)	0.015*
**18.5–24.9**	4,252 (18.3)	246 (21.1)	222 (20.1)	592 (22.7)	852 (19.9)	2,340 (16.6)	<0.001*
**25.0–29.9**	5,050 (21.7)	244 (21.0)	258 (23.4)	563 (21.6)	960 (22.4)	3,025 (21.4)	0.73
**> = 30.0**	6,468 (27.8)	248 (21.3)	254 (23.0)	515 (19.7)	1,056 (24.7)	4,395 (31.1)	<0.001*
**Missing**	7,074 (30.4)	414 (35.6)	349 (31.6)	899 (34.4)	1,330 (31.1)	4,082 (28.9)	<0.001*
**Comorbidities, No. (%)**							
**Hypertension**	7,725 (33.2)	273 (23.5)	315 (28.5)	661 (25.3)	1,307 (30.5)	5,169 (36.6)	<0.001*
**Diabetes**	4,395 (18.9)	122 (10.5)	154 (13.9)	320 (12.3)	699 (16.3)	3,100 (21.9)	<0.001*
**Coronary artery disease**	2,657 (11.4)	78 (6.7)	102 (9.2)	227 (8.7)	464 (10.8)	1,786 (12.6)	<0.001*
**Heart failure**	1,421 (6.1)	43 (3.7)	48 (4.3)	98 (3.8)	226 (5.3)	1,006 (7.1)	<0.001*
**COPD**	1,489 (6.4)	54 (4.6)	61 (5.5)	131 (5.0)	246 (5.7)	997 (7.1)	0.002*
**Asthma**	2,059 (8.8)	74 (6.4)	65 (5.9)	178 (6.8)	328 (7.7)	1,414 (10.0)	<0.001*
**Cancer**	2,972 (12.8)	138 (11.9)	138 (12.5)	293 (11.2)	568 (13.3)	1,835 (13.0)	0.27
**Obesity**	3,584 (15.4)	119 (10.2)	146 (13.2)	263 (10.1)	640 (14.9)	2,416 (17.1)	<0.001*
**Hyperlipidemia**	5,401 (23.2)	232 (19.9)	259 (23.4)	508 (19.4)	980 (22.9)	3,422 (24.2)	<0.001*
**Mortality, No. (%)**	1,920 (8.2)	35 (3.0)	71 (6.4)	131 (5.0)	338 (7.9)	1,345 (9.5)	0.001*

Notes

^a^P values were calculated by comparing patients from quintile 1 areas and those from quintile 5 areas using χ2 test for categorical variables or Wilcoxon rank-sum test for continuous variables. IQR: interquartile range.

* indicates FDR q-value < 0.05.

Compared to socially advantaged patients (SDI quintile 1), socially disadvantaged patients (SDI quintile 5) were more likely to be admitted to hospital (41.9% vs. 27.4%) and present to ED without hospitalization (21.3% vs. 7.0%). Socially disadvantaged patients were older (median age 55 vs. 53) and more likely to be male (48.6% vs. 44.2%). Although socially disadvantaged patients were more likely to be black or Hispanic than socially advantaged patients, they were racially and ethnically diverse. Only 23.1% of socially disadvantaged patients were black and 25.4% were Hispanic. In addition, socially disadvantaged patients were more likely to have higher BMI (median 28.9 vs. 27.2) and have multiple chronic conditions. For example, 36.6% of socially disadvantaged patients had hypertension and 21.9% had diabetes compared to only 23.5% and 10.5%, respectively, among socially advantaged patients. All these differences were statistically significant at the P<0.001 level.

The overall mortality was 8.2% among all patients. Socially disadvantaged patients had a mortality of 9.5%, more than three times higher than the mortality of socially advantaged patients (3.0%). All these differences were statistically significant at the P<0.001 level.

### Socioeconomic variation in inpatient characteristics and treatment

We identified 9,136 community-dwelling patients hospitalized for COVID-19. 19.2% of socially disadvantaged patients died in the hospital, more than double the mortality rate of socially advantaged patients (9.4%) (Table 2). Socially disadvantaged patients had presenting laboratory markers that indicated more severe disease, including higher venous lactate (median 1.7 vs. 1.5 mmol/L), white blood cell count (7.6 vs. 6.7 ×10^3^ cells/μL), platelet count (204 vs. 195 ×10^3^ cells/μL), and D-dimer (1.5 vs. 1.0 μg/mL), compared to socially advantaged patients (Table 2).

**Table 2:** Characteristics and treatment of hospitalized patients by quintiles of social deprivation index.

	Overall N = 9,136	Social Deprivation Index Quintiles					P value^a^
		Quintile 1 (socially advantaged) N = 309	Quintile 2 N = 386	Quintile 3 N = 851	Quintile 4 N = 1,668	Quintile 5 (socially disadvantaged) N = 5,922	
**Location prior to admission, No. (%)**							
**Facilities (e.g., other hospital)**	826 (9.0)	7 (2.3)	6 (1.6)	10 (1.2)	76 (4.6)	728 (12.3)	<0.001*
**Other (e.g., home)**	8,310 (91.0)	302 (97.7)	380 (98.4)	841 (98.8)	1,592 (95.4)	5,195 (87.7)	<0.001*
**Discharge status (%)**							
**Discharged alive**	7,526 (82.4)	280 (90.6)	324 (83.9)	744 (87.4)	1,392 (83.4)	4,786 (80.8)	<0.001*
**Died in hospital**	1,610 (17.6)	29 (9.4)	62 (16.1)	107 (12.6)	276 (16.6)	1,136 (19.2)	<0.001*
**Laboratory Results, median (IQR) and N**							
**Venous lactate (mmol/L)**	1.6 (1.2–2.3), 3,284	1.5 (1.1–1.9), 85	1.4 (1.0–1.9), 131	1.5 (1.2–1.9), 274	1.6 (1.1–2.1), 498	1.7 (1.2–2.4), 2,296	0.01*
**Creatinine (mg/dL)**	1.0 (0.8–1.6), 7,116	1.0 (0.8–1.4), 279	1.0 (0.7–1.3), 357	1.0 (0.8–1.4), 695	1.0 (0.8–1.5), 1,353	1.1 (0.8–1.7), 4,432	<0.001*
**White blood cell count (×10^3^ cells/μL)**	7.4 (5.4–10.2), 7,295	6.7 (4.7–9.5), 287	7.3 (5.5–9.7), 366	7.1 (5.3–9.8), 720	7.1 (5.2–10.1), 1,390	7.6 (5.6–10.4), 4,532	<0.001*
**Lymphocyte count (×10^3^ cells/μL)**	1.0 (0.7–1.4), 6,653	0.8 (0.6–1.2), 236	0.9 (0.6–1.3), 301	0.9 (0.6–1.3), 620	0.9 (0.6–1.3), 1,253	1.0 (0.7–1.4), 4,243	<0.001*
**Platelet count (×10^3^ cells/μL)**	204 (157–270), 7,277	195 (143–272), 287	210 (160–274), 365	207 (158–267), 719	204 (160–270), 1,387	204 (156–271), 4,519	0.12
**Bilirubin (mg/dL)**	0.4 (0.3–0.5), 6,721	0.5 (0.3–0.6), 258	0.4 (0.3–0.5), 333	0.4 (0.3–0.6), 642	0.4 (0.3–0.6), 1,275	0.4 (0.4–0.5), 4,213	<0.001*
**Aspartate aminotransferase (U/L)**	42 (28–67), 6,549	43 (30–65), 247	42 (31–61), 314	43 (28–66), 625	43 (28–70), 1,241	42 (28–67), 4,122	0.29
**Alanine aminotransferase (U/L)**	31 (20–52), 6,697	32 (21–52), 257	32 (22–48), 332	32 (19–54), 637	33 (21–56), 1,267	30 (19–50), 4,204	0.12
**Creatine kinase (U/L)**	156 (75–364), 4,188	153 (71–335), 165	142 (62–317), 203	141 (64–309), 388	154 (76–367), 749	162 (78–387), 2,683	0.24
**Prothrombin time (s)**	13.5 (12.4–14.8), 4,738	13.4 (12.3–15.1), 209	13.6 (12.4–15.3), 250	13.3 (12.3–14.6), 512	13.3 (12.2–14.6), 984	13.6 (12.6–14.8), 2,783	0.18
**Interleukin-6 (pg/mL)**	19 (9–47), 1,700	14 (8–33), 70	17 (9–40), 92	17 (9–45), 150	18 (9–36), 317	21 (9–50), 1,071	0.14
**C-reactive protein (mg/L)**	104 (47–173), 5,691	100 (54–157), 227	102 (49–162), 274	105 (43–170), 584	100 (48–161), 1,103	106 (47–178), 3,503	0.20
**Ferritin (ng/mL)**	663 (319–1,389), 5,429	619 (312–1,267), 208	612 (296–1218), 262	693 (303–1508), 557	658 (315–1,371), 1,054	665 (326–1403), 3,348	0.38
D-dimer (μg/mL)	1.3 (0.6–3.3), 1,710	1.0 (0.4–2.6), 52	0.7 (0.3–1.8), 47	0.7 (0.4–2.3) 113	1.0 (0.5–2.8), 228	1.5 (0.7–3.6), 1,270	0.01*
**Cardiac troponin T (ng/L)**	21 (10–70), 1,631	14 (9–33), 4	18 (8–50), 8	16 (9–44), 25	18 (10–60), 112	21 (10–70), 1,482	0.39
**Procalcitonin (ng/mL)**	0.2 (0.1–0.6), 4,444	0.2 (0.1–0.5), 174	0.3 0.1–0.7), 232	0.2 (0.1–0.5), 475	0.2 (0.1–0.5), 908	0.2 (0.1–0.6), 2,655	0.28
**Albumin (g/dl)**	3.6 (3.1–3.9), 6,777	3.7 (3.3–4.1), 263	3.8 (3.4–4.1), 343	3.7 (3.3–4.0), 654	3.5 (3.0–3.9), 1,288	3.5 (3.1–3.9), 4,229	<0.001*
**Red blood cell distribution width (%)**	13.8 (13.0–15.0), 7,292	13.7 (12.9–15.0), 287	13.6 (12.9–14.7), 366	13.7 (12.9–14.8), 720	13.8 (13.0–15.0), 1,388	13.9 (13.1–15.1), 4,531	0.06
**Neutrophil count (×10^3^ cells/μL)**	5.6 (3.9–8.2), 6,635	5.2 (3.5–7.7), 236	5.3 (3.8–7.6), 301	5.3 (3.7–7.8), 621	5.4 (3.7–8.0), 1,248	5.8 (4.0–8.4), 4,229	0.004*
**Treatment and procedures**							
**Length of stay, median (IQR)**	7 (4–12)	7 (4–14)	8 (4–12)	7 (4–13)	7 (4–12)	7 (4–12)	0.20
**ICU care, No. (%)**	1,302 (14.3)	62 (20.1)	76 (19.7)	146 (17.2)	310 (18.6)	708 (12.0)	<0.001*
**Invasive mechanical ventilation, No. (%)**	1,301 (14.4)	31 (10.0)	53 (13.7)	103 (12.1)	259 (15.5)	855 (14.4)	0.03
**Respiratory Ventilation, Less than 24 Consecutive Hours, No. (%)**	162 (1.8)	4 (1.3)	5 (1.3)	11 (1.3)	28 (1.7)	114 (1.9)	0.43
**Respiratory Ventilation, 24–96 Consecutive Hours, No. (%)**	230 (2.5)	2 (0.7)	9 (2.3)	19 (2.2)	44 (2.6)	156 (2.6)	0.03
**Respiratory Ventilation, Greater than 96 Consecutive Hours, No. (%)**	785 (8.6)	25 (8.1)	39 (10.1)	71 (8.3)	180 (10.8)	470 (7.9)	0.95
**Renal replacement therapy, No. (%)**	393 (4.3)	12 (3.9)	16 (4.2)	28 (3.3)	72 (4.3)	265 (4.5)	0.63
**Vasopressor use, No. (%)**	1,201 (13.2)	40 (12.9)	61 (15.8)	118 (13.9)	259 (15.5)	723 (12.2)	0.72
**Hydroxychloroquine, No. (%)**	2,050 (22.4)	10 (3.2)	11 (2.9)	86 (10.1)	197 (11.6)	1,750 (29.6)	<0.001*
**Steroid, No. (%)**	1,538 (16.8)	41 (13.3)	54 (14.0)	121 (14.2)	231 (13.9)	1,091 (18.4)	<0.001*
**Discharge destinations, No. (%)**							
** Home**	3,510 (46.6)	77 (27.5)	102 (31.5)	325 (43.7)	612 (44.0)	2,394 (50.0)	<0.001*
** Hospice**	192 (2.6)	7 (2.5)	8 (2.5)	22 (3.0)	44 (3.2)	111 (2.3)	0.84
** Other acute inpatient hospital**	23 (0.3)	1 (0.4)	1 (0.3)	3 (0.4)	4 (0.3)	14 (0.3)	0.84
** Long-term care facilities/rehab**	1,943 (25.8)	91 (32.5)	112 (34.5)	160 (21.5)	350 (25.1)	1,230 (25.7)	0.008*
** Other**	1,858 (24.7)	104 (37.1)	101 (31.2)	234 (31.5)	382 (27.4)	1,037 (21.7)	<0.001*

Notes

^a^ P values were calculated by comparing patients from quintile 1 areas and those from quintile 5 areas using χ2 test for categorical variables or Wilcoxon rank-sum test for continuous variables. IQR: interquartile range.

* indicates FDR q-value < 0.05.

Socially disadvantaged patients were more likely to receive mechanical ventilation (14.4% vs. 10.0%), renal replacement therapy (4.5% vs. 3.9%), and hydroxychloroquine (29.6% vs. 3.2%), compared to socially advantaged patients (Table 2). Finally, socially disadvantaged patients were more likely to be discharged home (50.0% vs. 27.4%) and less likely to be discharged to hospice or long-term care facilities (25.7% vs. 32.5%), as compared to socially advantaged patients (Table 2).

### Socioeconomic variation in healthcare utilization

As compared to socially advantaged patients, socially disadvantaged patients were more than twice as likely to have their first COVID-19 encounter in the ED (57.4% vs. 26.1%) than in ambulatory clinics. Overall, among 7,526 hospitalized community dwelling patients who were discharged alive, 5.9% of them had re-hospitalizations within 30 days of discharge, 4.6% had ED visits, and 36.8% had ambulatory visits (Table 3). Socially disadvantaged patients had higher rates of post-discharge healthcare utilization (Table 3). For example, 6.3% of socially disadvantaged patients had a re-hospitalization within 30 days after discharge, as compared to 3.2% of socially advantaged patients.

**Table 3:** Healthcare utilization 30-day after discharge by quintiles of social deprivation index.

		Social Deprivation Index Quintiles					
Hospitalized patients, No. (%)	Overall ^a^ N = 7,526	Quintile 1 N = 280	Quintile 2 N = 324	Quintile 3 N = 744	Quintile 4 N = 1,392	Quintile 5 N = 4,786	P value ^a^
**Any ambulatory visits**	2,771 (36.8)	78 (27.9)	79 (24.4)	250 (33.6)	464 (33.3)	1,900 (39.7)	<0.001*
**Any emergency department visits**	347 (4.6)	9 (3.2)	11 (3.4)	35 (4.7)	54 (3.9)	238 (5.0)	0.17
**Any hospitalizations**	444 (5.9)	9 (3.2)	18 (5.6)	48 (6.5)	70 (5.0)	299 (6.3)	0.03*
**Patients presented to ED without hospitalization, No. (%)**	Overall ^a^ N = 3,851	Quintile 1 N = 80	Quintile 2 N = 101	Quintile 3 N = 275	Quintile 4 N = 454	Quintile 5 N = 2,941	P value ^a^
**Any ambulatory visits**	1,012 (26.4)	20 (25.0)	25 (24.8)	75 (27.3)	112 (24.7)	780 (26.5)	0.90
**Any emergency department visits**	459 (11.9)	16 (20.0)	11 (10.9)	33 (12.0)	46 (10.1)	353 (12.0)	0.03
**Any hospitalizations**	11 (0.3)	0 (0.0)	1 (1.0)	1 (0.4)	0 (0.0)	9 (0.3)	>0.99
**Patients presented to ambulatory clinics only, No. (%)**	Overall ^a^ N = 10,226	Quintile 1 N = 760	Quintile 2 N = 609	Quintile 3 N = 1,507	Quintile 4 N = 2,143	Quintile 5 N = 5,207	P value ^a^
**Any ambulatory visits**	4,786 (46.8)	307 (40.4)	227 (37.3)	602 (40.0)	977 (45.6)	2,673 (51.3)	<0.001 *
**Any emergency department visits**	62 (0.6)	4 (0.5)	6 (1.0)	5 (0.3)	10 (0.5)	37 (0.7)	0.57
**Any hospitalizations**	26 (0.3)	0 (0.0)	2 (0.3)	1 (0.1)	3 (0.1)	20 (0.4)	0.10

Notes: This analysis only include patients who were discharged alive.

^a^ P values were calculated by comparing patients from quintile 1 areas (socially advantaged) and those from quintile 5 areas (socially disadvantaged) using χ2 test for categorical variables or Wilcoxon rank-sum test for continuous variables.

* indicates FDR q-value < 0.05

### Association of social conditions with hospitalization and mortality

**Fig 2:**
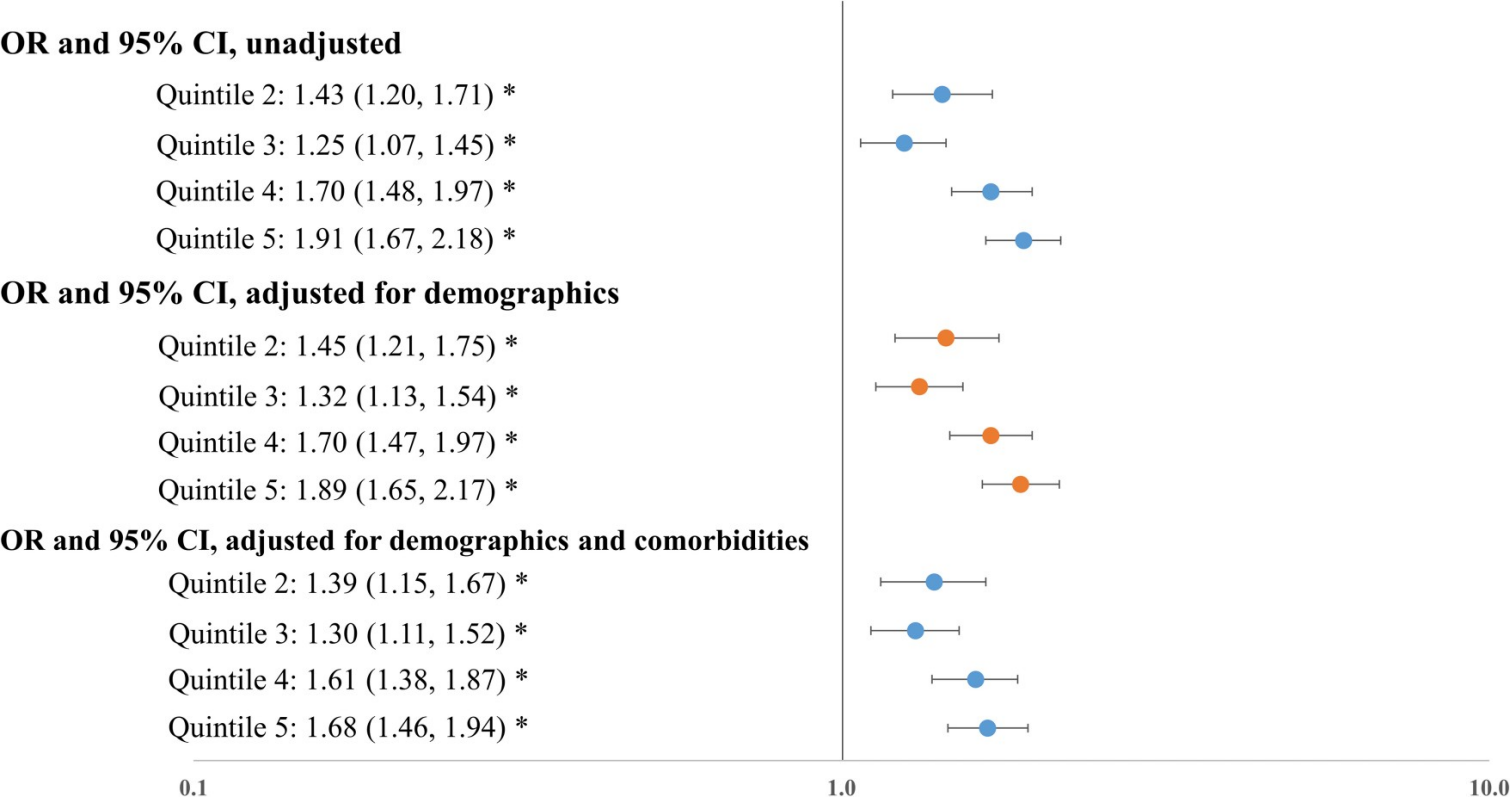
Association between social deprivation index quintiles and hospitalization. Notes: OR: odds ratio. Results were obtained from logistic regressions where hospitalization was the outcome. Demographics include age, gender, race, ethnicity; comorbidities include hypertension, diabetes, coronary artery disease, heart failure, COPD, asthma, cancer, obesity, and hyperlipidemia. * indicates that FDR q-value < 0.05.

**Fig 3:**
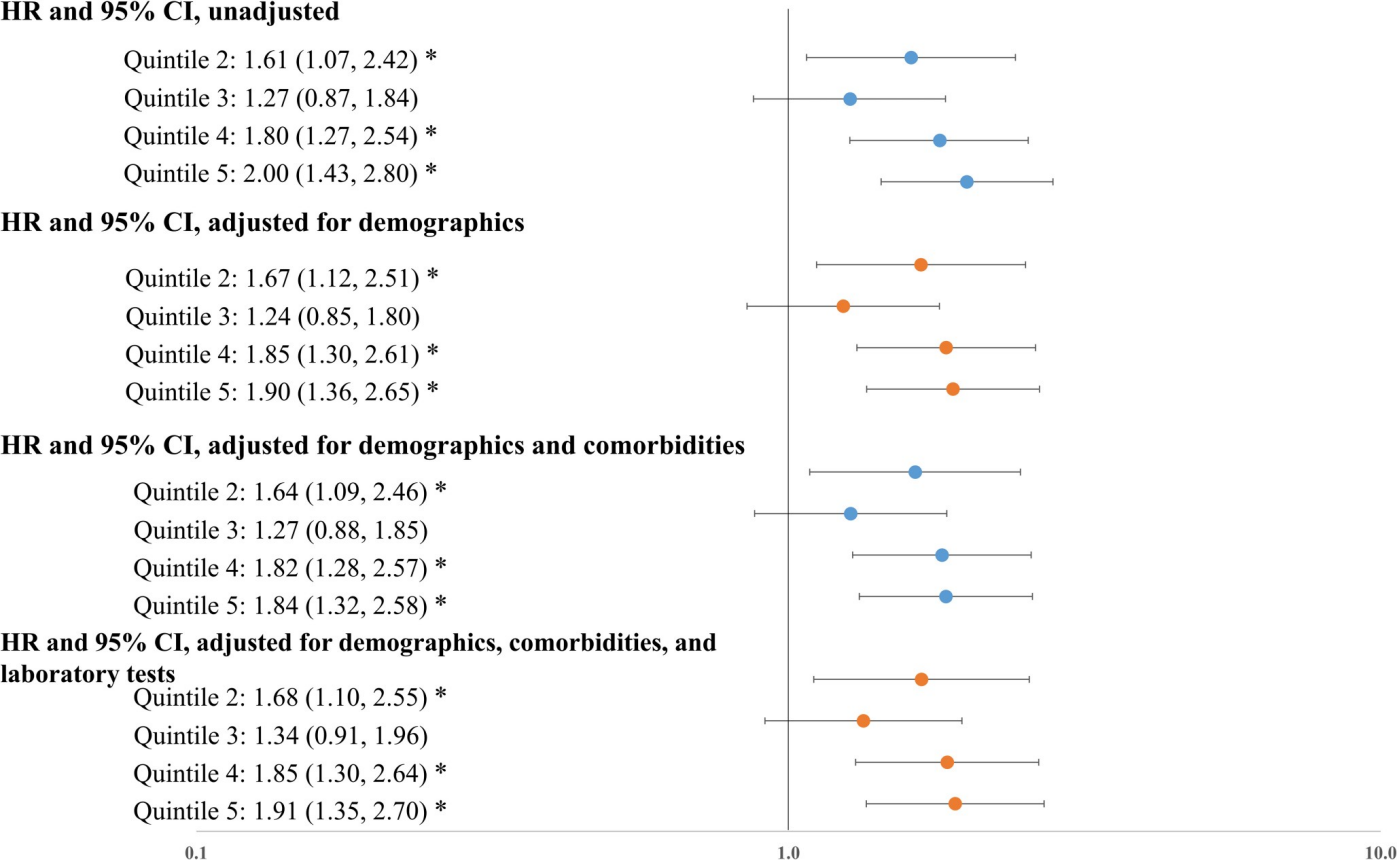
Association between social deprivation index quintiles and mortality. Notes: HR: hazard ratio. Results were obtained from Cox proportional-hazards models where death was the outcome. Demographics include age, gender, race, ethnicity; comorbidities include hypertension, diabetes, coronary artery disease, heart failure, COPD, asthma, cancer, obesity, and hyperlipidemia. Laboratory tests include indicators of high creatinine (>1.5 mg/dL), low white blood cell count (< 4×103 cells/μL), high white blood cell count (> 10×103 cells/μL), low lymphocyte count (< 1×103 cells/μL), low platelet count (<150 ×103 cells/μL), high bilirubin (≥ 1.2 mg/dL), high aspartate aminotransferase (> 40 U/L), low albumin (< 3.5 g/dl), high red blood cell distribution width (> 13.5%), and high neutrophil count (> 7.4 ×103 cells/μL).

Disadvantaged social conditions were associated with increased risk of hospitalization and mortality. Without adjusting for patient characteristics, socially disadvantaged patients were almost two times as likely to be hospitalized when compared to socially advantaged patients (odds ratio [OR]: 1.91, P<0.001). This association remained statistically significant after adjusting for patient demographics (OR: 1.89, P<0.001) and for demographics and baseline comorbidities (OR: 1.68, P<0.001) (Fig 2). Similarly, the unadjusted Cox model showed that socially disadvantaged patients were twice as likely to die when compared to socially advantaged patients (hazard ratio (HR): 2.00, P<0.001). Adjusting for patient demographics, baseline comorbidities, and presenting laboratory test results produced similar results (HRs ranged from 1.84 to 1.91) (Fig 3). Full regression results are available in the appendix (S1 and S2 Tables). We verified that the variance inflation factor (VIF) in these models for each covariate was below 10, indicating a low level of multi-collinearity.

### Secondary and sensitivity analyses

The adjusted logistic regressions and Cox models showed that all the five measures of social conditions, including income, education, occupation, housing conditions, and unemployment, had statistically significant associations with hospitalization and mortality (S3 and S4 Tables).

Among 4,577 long-term care facility residents, 4,152 (90.7%) were from neighborhoods with disadvantaged social conditions. Most of these patients (97.1%) were hospitalized. Compared to community dwelling patients, long-term care facility residents were older, more likely to be underrepresented minorities, and more likely to have chronic conditions (S5–S7 Tables).

## Discussion

To our knowledge, this is among the first and largest studies to describe the relationship between socioeconomic vulnerabilities, clinical outcomes, and healthcare utilization for COVID-19 patients across multiple care settings in NYC, a major epicenter of the coronavirus pandemic in the United States. We found substantial variation in patient characteristics, health outcomes, and healthcare utilization by social conditions. While patients with the highest level of social disadvantage comprised one-third of the NYC population, they accounted for over 60% of COVID-19 patients. Compared to other patients, social disadvantaged patients were more likely to be hospitalized, to present to the ED without being hospitalized, and were more than three times as likely to die of COVID-19.

Using aggregated regional level data, prior studies have found evidence of higher rates of infection, hospitalization, and mortality in socially vulnerable areas [1, 36, 37]. Using county-level data, a recent study found that social risk factors are associated with increased COVID-19 incidence and mortality [38]. However, no studies have linked social condition data with detailed patient-level clinical data. We extend these studies by linking neighborhood social data with granular clinical data and find that patients with disadvantaged social conditions have significantly different demographics, clinical conditions, and presenting laboratory test results, suggesting that they may have presented to care later in the disease course or with more severe disease. Our findings provide distinct and unique evidence that may be relevant for improving health outcomes among socially vulnerable patients.

The reasons for the observed socioeconomic disparities require further study. Patients in areas with high social disadvantage have poorer baseline health status and other risk factors for severe COVID-19, including older age, male gender, and racial/ethnic minority status. These patients may also have presented to care later in their disease course. Among hospitalized patients, socially disadvantaged patients presented with more severe disease markers, such as elevated venous lactate and white blood cell count. Disadvantaged neighborhood social conditions were associated with a significantly increased risk of hospitalization and mortality, after adjusting for race, ethnicity, and other patient demographic and clinical characteristics. Understanding the reasons for these disparities may inform prevention and treatment strategies for COVID-19 and other diseases to promote health equity.

Many drivers of social vulnerability, including food insecurity, poor housing conditions, and limited access to technology (e.g., internet) may be relevant to poor outcomes for COVID-19 patients [22, 39–41]. For example, essential workers are not able to work from home, placing them at higher risk for infection [42]. School closures have increased levels of food insecurity for children living in poverty, which is associated with malnutrition and higher risk of coronavirus infection and transmission to family members [43]. Patients with poor health literacy may be less likely to appreciate the need for social distancing and other precautionary measures during the COVID-19 pandemic [44, 45]. In addition, self-quarantine may be not feasible for patients living in crowded home environments [44]. Future studies are needed to examine the contribution of these factors at individual patient level to adverse COVID-19 outcomes. As the pandemic evolves, better meeting the social, economic, and health needs of socially disadvantaged is needed to help reduce disparities.

Social vulnerabilities and racial/ethnic disparities are related but distinct [20, 46, 47], a finding further substantiated by our analyses. A relatively high proportion of patients from the most socially disadvantaged neighborhoods were not from racial/ethnic minority groups. Furthermore, in our study social disadvantage had an independent and statistically significant association with higher rates of hospitalization and mortality, suggesting that addressing inequities in COVID-19 outcomes may require interventions that focus broadly on socially disadvantaged populations. Neighborhood-based policies in which resources are determined by differential disease rates may offer an important avenue to target support, as communities contend with both the acute and chronic effects of the COVID-19 pandemic. NYC previously used such a micro-cluster strategy for implementing regulations and closures in areas with higher rates of COVID-19 infection [48].

In addition to relatively poor clinical outcomes among socially disadvantaged patients, we found that such patients were more likely to receive intensive treatment during the hospitalization, which is consistent with higher severity of disease as measured by laboratory tests at presentation. In our study and in previous research, socially disadvantaged patients were more likely to have chronic conditions, such as diabetes, COPD, and obesity, which may contribute to more severe illness and more intensive treatment during hospitalization [21, 49, 50]. It is also possible that socially disadvantaged patients presented to care later in the disease course, due to limited access to transportation, lack of health insurance, or fewer healthcare resources in their neighborhoods [44, 51].

A particularly important contribution of this study is that it examined downstream healthcare utilization, by social conditions, after an ED visit or hospital admission for COVID-19. The lingering multi-organ sequelae of COVID-19 after the acute illness—sometimes called “long COVID”—are increasingly being recognized, and include adverse effects for cardiovascular health, mental health, and activities of daily living [52–54]. Hospital readmission and other health care utilization are common after acute COVID-19 [55, 56]. We found that socially disadvantaged patients were more likely to experience healthcare utilization after an ED visit or hospital admission, indicating that socially disadvantaged patients may be disproportionately affected by the medium- and long-term sequelae of COVID-19. This may be due to higher pre-existing levels of chronic conditions, vulnerable social conditions (e.g., food insecurity), or the interaction between medical and social conditions. Focused attention and dedicated interventions are needed to improve health outcomes after acute COVID-19, particularly for socially disadvantaged patients.

Taken together, the findings of this study make clear that ensuring equitable access to COVID-19 vaccination should be a priority for the U.S. COVID-19 vaccination program. Early evidence suggests that vaccination has been lower among residents of counties with disadvantaged social conditions [57, 58]. This may be due to limited vaccination supply, difficulty taking time off from work, or higher rates of mistrust of the medical system and vaccine hesitancy [59]. More research is warranted to understand the social barriers to improve the vaccination coverage.

This study has several limitations. First, although we draw on the largest COVID-19 patient cohort from five health systems in NYC, findings may not be generalizable to other patients in the NYC area or patients in other parts of the country. Second, our analysis was limited to outcomes and utilization occurring within these health systems; clinical encounters at other health systems that were not included. Therefore, it is possible that healthcare utilization after acute COVID-19 was underestimated. Third, we were not able to extract details of presenting symptoms, as these measures are generally coded in a non-standardized way. Similarly, we were not able to examine some risk factors, such as Vitamin D deficiency, as they were not routinely tested in early in the pandemic. In addition, we may have underestimated ICU admissions because during the pandemic, non-ICU nursing units were converted to ICUs to accommodate the larger volume of critically ill patients. Finally, we examined social conditions at the zip code level; using more granular data, such as data at the US census block group level, could further characterize patient social conditions. Similarly, detailed social condition data at individual patient level, such as health literacy, food access, and living environment, could elucidate the impact of these factors on socioeconomic disparities related to COVID-19.

## Conclusion

We found substantial variation in characteristics, outcomes, and healthcare utilization by neighborhood social conditions among COVID-19 patients in NYC. Individuals affected by COVID-19 were disproportionately from neighborhoods with disadvantaged social conditions. These patients were at higher risk for hospitalization and mortality, after adjusting for other patient characteristics, including race and ethnicity. In addition, patients with disadvantaged social conditions received more intensive treatment during hospitalization and were more likely to require medical care after treatment for acute COVID-19. Health care leaders, policymakers, and public health practitioners should consider prioritizing socially disadvantaged areas when designing interventions or allocating resources to reduce health disparities related to COVID-19.

## Supporting information

S1 Table Results of logistic regressions for examining the association between SDI quintiles and hospitalization. (DOCX)

S2 Table Results of Cox models for examining the association between SDI quintiles and mortality. (DOCX)

S3 Table Adjusted associations between neighborhood social conditions and hospitalization, logistic regressions. (DOCX)

S4 Table Adjusted associations between neighborhood social conditions and mortality, Cox models. (DOCX)

S5 Table Overall patient characteristics by quintiles of social deprivation index, long-term care facility residents. (DOCX)

S6 Table Characteristics and treatment of hospitalized patients by quintiles of social deprivation index, long-term care facility residents. (DOCX)

S7 Table Healthcare utilization 30-day after discharge by quintiles of social deprivation index, long-term care facility residents. (DOCX)
