# Supplementary material for: Socioeconomic variation in characteristics, outcomes, and healthcare utilization of COVID-19 patients in New York City
Source: PLoS One. 2021 Jul 29;16(7):e0255171. doi: 10.1371/journal.pone.0255171 (PMC8321227; doi:10.1371/journal.pone.0255171)
Supplement: S1 Table — (DOCX) [file pone.0255171.s001.docx]

# **S1 Table. Results of Logistic Regressions for Examining the Association between SDI Quintiles and Hospitalization**

|  | Model 1 | Model 2 | Model 3 |
| --- | --- | --- | --- |
|  | **Odds ratio (95% CI), P value** | | |
| SDI quintiles |  |  |  |
| Quintile 1 (Ref.) | 1.00 | 1.00 | 1.00 |
| Quintile 2 | 1.43 (1.20, 1.71), <0.001 * | 1.45 (1.21, 1.75), <0.001 * | 1.39 (1.15, 1.67), 0.001 * |
| Quintile 3 | 1.25 (1.07, 1.45), 0.004 * | 1.32 (1.13, 1.54), <0.001 * | 1.30 (1.11, 1.52), 0.001 * |
| Quintile 4 | 1.70 (1.48, 1.97), <0.001 * | 1.70 (1.47, 1.97), <0.001 * | 1.61 (1.38, 1.87), <0.001 * |
| Quintile 5 | 1.91 (1.67, 2.18), <0.001 * | 1.89 (1.65, 2.17), <0.001 * | 1.68 (1.46, 1.94), <0.001 * |
| Age |  | 1.04 (1.04, 1.05), <0.001 * | 1.03 (1.03, 1.04), <0.001 * |
| Gender |  |  |  |
| Female (ref.) |  | 1.00 | 1.00 |
| Male |  | 1.59 (1.50, 1.68), <0.001 | 1.55 (1.46, 1.65), <0.001 |
| Other/Unknown |  | -- | -- |
| Race |  |  |  |
| White (ref.) |  | 1.00 | 1.00 |
| Black |  | 0.96 (0.88, 1.04), 0.32 | 0.76 (0.70, 0.84), <0.001 |
| Asian |  | 1.04 (0.91, 1.18), 0.59 | 1.07 (0.94, 1.23), 0.31 |
| Other/unknown |  | 1.06 (0.98, 1.14), 0.17 | 0.99 (0.92, 1.08), 0.88 |
| Ethnicity |  |  |  |
| Hispanic (ref.) |  | 1.00 | 1.00 |
| Non-Hispanic |  | 0.94 (0.86, 1.03), 0.19 | 0.99 (0.90, 1.08), 0.77 |
| Unknown |  | 0.56 (0.51, 0.61), <0.001 | 0.66 (0.60, 0.72), <0.001 |
| Comorbidities |  |  |  |
| Hypertension |  |  | 1.62 (1.49, 1.75), <0.001 |
| Diabetes |  |  | 1.88 (1.72, 2.06), <0.001 |
| Coronary artery disease |  |  | 1.46 (1.30, 1.64), <0.001 |
| Heart failure |  |  | 1.89 (1.61, 2.21), <0.001 |
| COPD |  |  | 1.01 (0.89, 1.15),  0.86 |
| Asthma |  |  | 0.99 (0.88, 1.11), 0.85 |
| Cancer |  |  | 0.71 (0.65, 0.78), <0.001 |
| Obesity |  |  | 1.83 (0.92, 1.09),  <0.001 |
| Hyperlipidemia |  |  | 1.00 (0.92, 1.09),  0.99 |

** indicates FDR q-value < 0.05.*
