# Supplementary material for: Socioeconomic variation in characteristics, outcomes, and healthcare utilization of COVID-19 patients in New York City
Source: PLoS One. 2021 Jul 29;16(7):e0255171. doi: 10.1371/journal.pone.0255171 (PMC8321227; doi:10.1371/journal.pone.0255171)
Supplement: S2 Table — (DOCX) [file pone.0255171.s002.docx]

# **S2 Table. Results of Cox Models for Examining the Association between SDI Quintiles and Mortality**

|  | Model 1 | Model 2 | Model 3 | Model 4 |  |
| --- | --- | --- | --- | --- | --- |
|  | **Hazard ratio (95% CI), P value** | | | |  |
| SDI quintiles |  |  |  |  |  |
| Quintile 1 (Ref.) | 1.00 | 1.00 | 1.00 | 1.00 |  |
| Quintile 2 | 1.61 (1.07, 2.42), 0.02 * | 1.67 (1.12, 2.51), 0.01 * | 1.64 (1.09, 2.46), 0.02 * | 1.68 (1.10, 2.55), 0.02 * |  |
| Quintile 3 | 1.27 (0.87, 1.84), 0.21 | 1.24 (0.85, 1.80), 0.27 | 1.27 (0.88, 1.85), 0.21 | 1.34 (0.91, 1.96), 0.14 |  |
| Quintile 4 | 1.80 (1.27, 2.54), 0.001 * | 1.85 (1.30, 2.61), 0.001 * | 1.82 (1.28, 2.57), 0.001 * | 1.85 (1.30, 2.64), 0.001 * |  |
| Quintile 5 | 2.00 (1.43, 2.80), <0.001 * | 1.90 (1.36, 2.65), <0.001 * | 1.84 (1.32, 2.58), <0.001 * | 1.91 (1.35, 2.70), <0.001 * |  |
| Age |  | 1.05 (1.04, 1.05), <0.001 | 1.04 (1.04, 1.05), <0.001 | 1.04 (1.03, 1.04), <0.001 |  |
| Gender |  |  |  |  |  |
| Female (ref.) |  | 1.00 | 1.00 | 1.00 |  |
| Male |  | 1.37 (1.24, 1.51), <0.001 | 1.36 (1.23, 1.50), <0.001 | 1.19 (1.07, 1.32), 0.001 |  |
| Other/Unknown | -- | -- | -- | -- | -- |
| Race |  |  |  |  |  |
| White (ref.) |  | 1.00 | 1.00 | 1.00 |  |
| Black |  | 1.10 (0.93, 1.26), 0.16 | 1.04 (0.91, 1.19), 0.55 | 1.02 (0.89, 1.18), 0.78 |  |
| Asian |  | 0.96 (0.76, 1.21), 0.75 | 1.09 (0.86, 1.37), 0.48 | 0.96 (0.76, 1.21), 0.73 |  |
| Other/unknown |  | 0.91 (0.81, 1.03), 0.14 | 0.90 (0.79, 1.02), 0.09 | 0.94 (0.83, 1.07), 0.33 |  |
| Ethnicity |  |  |  |  |  |
| Hispanic (ref.) |  | 1.00 | 1.00 | 1.00 |  |
| Non-Hispanic |  | 0.64 (0.56, 0.73), <0.001 | 0.60 (0.53, 0.69), <0.001 | 0.69 (0.60, 0.79), <0.001 |  |
| Unknown |  | 0.97 (0.85, 1.11), 0.69 | 1.00 (0.88, 1.15), 0.97 | 1.23 (1.07, 1.41), 0.004 |  |
| Comorbidities |  |  |  |  |  |
| Hypertension |  |  | 1.12 (0.99, 1.28), 0.08 | 1.20 (1.05, 1.37), 0.007 |  |
| Diabetes |  |  | 1.26 (1.13, 1.41), <0.001 | 1.28 (1.14, 1.43), <0.001 |  |
| Coronary artery disease |  |  | 1.13 (0.99, 1.27), 0.06 | 1.10 (0.97, 1.25), 0.13 |  |
| Heart failure |  |  | 1.42 (1.24, 1.62), <0.001 | 1.40 (1.22, 1.60), <0.001 |  |
| COPD |  |  | 1.05 (0.91, 1.21), 0.48 | 1.07 (0.93, 1.24), 0.33 |  |
| Asthma |  |  | 0.87 (0.74, 1.02), 0.09 | 0.91 (0.77, 1.07), 0.25 |  |
| Cancer |  |  | 1.12 (0.99, 1.26), 0.06 | 1.10 (0.97, 1.24), 0.14 |  |
| Obesity |  |  | 1.35 (1.21, 1.52), <0.001 | 1.28 (1.13, 1.44), <0.001 |  |
| Hyperlipidemia |  |  | 0.99 (0.88, 1.11), 0.85 | 0.98 (0.87, 1.11), 0.73 |  |
| Laboratory tests |  |  |  |  |  |
| Creatinine >1.5 mg/dL |  |  |  | 1.36 (1.22, 1.52), <0.001 |  |
| White blood cell count < 4×10^3^ cells/μL |  |  |  | 0.75 (0.60, 0.94), 0.01 |  |
| White blood cell count > 10×10^3^ cells/μL |  |  |  | 1.26 (1.05, 1.52), 0.01 |  |
| Lymphocyte count < 1×10^3^ cells/μL |  |  |  | 1.09 (0.98, 1.21), 0.10 |  |
| Platelet count <150 ×10^3^ cells/μL |  |  |  | 1.30 (1.15, 1.47), <0.001 |  |
| Bilirubin ≥ 1.2 mg/dL |  |  |  | 1.32 (1.03, 1.71), 0.03 |  |
| Aspartate aminotransferase > 40 U/L |  |  |  | 1.53 (1.39, 1.69), <0.001 |  |
| Albumin < 3.5 g/dl |  |  |  | 1.15 (1.04, 1.27), 0.008 |  |
| Red blood cell distribution width > 13.5% |  |  |  | 0.93 (0.84, 1.03), 0.17 |  |
| Neutrophil count > 7.4 ×10^3^ cells/μL |  |  |  | 1.25 (1.04, 1.50),  0.02 |  |

** indicates FDR q-value < 0.05*
