# Supplementary material for: Socioeconomic variation in characteristics, outcomes, and healthcare utilization of COVID-19 patients in New York City
Source: PLoS One. 2021 Jul 29;16(7):e0255171. doi: 10.1371/journal.pone.0255171 (PMC8321227; doi:10.1371/journal.pone.0255171)
Supplement: S3 Table — (DOCX) [file pone.0255171.s003.docx]

# **S3 Table. Adjusted Associations between Neighborhood Social Conditions and Hospitalization, Logistic Regressions**

| Odds ratio (95% CI), P value | | | | | |
| --- | --- | --- | --- | --- | --- |
| Quintiles of social condition measures | Low income ^a^ | Education | Essential workers | Unemployment rate | Crowding housing |
| Quintile 1 (Ref.) | 1.00 | 1.00 | 1.00 | 1.00 | 1.00 |
| Quintile 2 | 1.27 (1.11, 1.44), 0.001 * | 0.89 (0.76, 1.05), 0.16 | 1.05 (0.93, 1.19), 0.46 | 1.42 (1.25, 1.62), <0.001 * | 1.41 (1.11, 1.80), 0.004 * |
| Quintile 3 | 1.16 (1.01, 1.33), 0.04 | 1.38 (1.18, 1.61), <0.001 * | 1.17 (1.04, 1.31), 0.007 * | 1.54 (1.35, 1.76), <0.001 * | 1.49 (1.20, 1.84), <0.001 * |
| Quintile 4 | 1.71 (1.52, 1.91), <0.001 * | 1.32 (1.16, 1.50), <0.001 * | 1.55 (1.41, 1.71), <0.001 * | 1.66 (1.47, 1.87), <0.001 * | 1.36 (1.13, 1.63), 0.001 * |
| Quintile 5 | 1.32 (1.18, 1.47), <0.001 * | 1.62 (1.44, 1.82), <0.001 * | 1.04 (0.92, 1.11), 0.76 | 1.25 (1.11, 1.41), <0.001 * | 2.02 (1.69, 2.42), <0.001 * |
| Age | 1.03 (1.03, 1.04), <0.001 | 1.03 (1.03, 1.04), <0.001 | 1.03 (1.03, 1.04), <0.001 | 1.03 (1.03, 1.03), <0.001 | 1.03 (1.03, 1.04), <0.001 |
| Gender |  |  |  |  |  |
| Female (ref.) | 1.00 | 1.00 | 1.00 | 1.00 | 1.00 |
| Male | 1.56 (1.47, 1.66), <0.001 | 1.56 (1.49, 1.66), <0.001 | 1.57 (1.47, 1.66), <0.001 | 1.55 (1.46, 1.65), <0.001 | 1.55 (1.46, 1.65), <0.001 |
| Other/Unknown | -- | -- | -- | -- | -- |
| Race |  |  |  |  |  |
| White (ref.) | 1.00 | 1.00 | 1.00 | 1.00 | 1.00 |
| Black | 0.79 (0.72, 0.87), <0.001 | 0.74 (0.68, 0.81), <0.001 | 0.85 (0.77, 0.93), <0.001 | 0.84 (0.76, 0.92), <0.001 | 0.75 (0.69, 0.82), <0.001 |
| Asian | 1.08 (0.94, 1.24), 0.28 | 1.06 (0.92, 1.22), 0.42 | 1.11 (0.96, 1.28), 0.15 | 1.09 (0.95, 1.26), 0.21 | 1.07 (0.93, 1.23), 0.35 |
| Other/unknown | 1.01 (0.93, 1.10), 0.78 | 0.98 (0.91, 1.06), 0.66 | 1.05 (0.97, 1.14), 0.19 | 1.05 (0.97, 1.13), 0.27 | 0.98 (0.90, 1.06), 0.63 |
| Ethnicity |  |  |  |  |  |
| Hispanic (ref.) | 1.00 | 1.00 | 1.00 | 1.00 | 1.00 |
| Non-Hispanic | 0.97 (0.88, 1.06), 0.48 | 1.04 (0.95, 1.14), 0.43 | 0.95 (0.87, 1.05), 0.32 | 0.93 (0.85, 1.01), 0.10 | 1.02 (0.93, 1.12), 0.62 |
| Unknown | 0.65 (0.60, 0.72), <0.001 | 0.69 (0.63, 0.75), <0.001 | 0.63 (0.58, 0.69), <0.001 | 0.64 (0.58, 0.69), <0.001 | 0.68 (0.62, 0.75), <0.001 |
| Comorbidities |  |  |  |  |  |
| Hypertension | 1.63 (1.50, 1.77), <0.001 | 1.60 (1.48, 1.74), <0.001 | 1.63 (1.50, 1.76), <0.001 | 1.64 (1.51, 1.78), <0.001 | 1.59 (1.47, 1.73), <0.001 |
| Diabetes | 1.88 (1.72, 2.06), <0.001 | 1.87 (1.71, 2.04), <0.001 | 1.90 (1.74, 2.08), <0.001 | 1.90 (1.74, 2.08), <0.001 | 1.86 (1.71, 2.03), <0.001 |
| Coronary artery disease | 1.47 (1.31, 1.64), <0.001 | 1.47 (1.32, 1.65), <0.001 | 1.48 (1.32, 1.65), <0.001 | 1.47 (1.31, 1.65), <0.001 | 1.46 (1.30, 1.64), <0.001 |
| Heart failure | 1.89 (1.62, 2.21), <0.001 | 1.86 (1.60, 2.17), <0.001 | 1.90 (1.63, 2.21), <0.001 | 1.91 (1.64, 2.23), <0.001 | 1.88 (1.62, 2.20), <0.001 |
| COPD | 1.02 (0.89, 1.16),  0.82 | 1.01 (0.88, 1.15),  0.90 | 1.02 (0.89, 1.16),  0.79 | 1.02 (0.90, 1.17),  0.73 | 1.01 (0.88, 1.15),  0.90 |
| Asthma | 0.99 (0.89, 1.11), 0.97 | 0.99 (0.89, 1.11), 0.89 | 0.99 (0.89, 1.11), 0.92 | 1.00 (0.90, 1.12), 0.96 | 0.99 (0.89, 1.11), 0.86 |
| Cancer | 0.71 (0.65, 0.78), <0.001 | 0.71 (0.65, 0.78), <0.001 | 0.72 (0.66, 0.79), <0.001 | 0.72 (0.65, 0.78), <0.001 | 0.72 (0.65, 0.79), <0.001 |
| Obesity | 1.82 (1.67, 1.99),  <0.001 | 1.81 (1.66, 1.98),  <0.001 | 1.84 (1.69, 2.01),  <0.001 | 1.82 (1.66, 1.98),  <0.001 | 1.82 (1.67, 1.99),  <0.001 |
| Hyperlipidemia | 0.99 (0.91, 1.08),  0.88 | 0.99 (0.92, 1.09),  0.95 | 1.01 (0.92, 1.10),  0.89 | 0.98 (0.90, 1.07),  0.73 | 0.99 (0.91, 1.08),  0.90 |

*Notes: SDI: Social Deprivation Index. ^a^ For income, higher quintiles indicate lower income. * indicates FDR q-value < 0.05.*
