# Supplementary material for: Socioeconomic variation in characteristics, outcomes, and healthcare utilization of COVID-19 patients in New York City
Source: PLoS One. 2021 Jul 29;16(7):e0255171. doi: 10.1371/journal.pone.0255171 (PMC8321227; doi:10.1371/journal.pone.0255171)
Supplement: S4 Table — (DOCX) [file pone.0255171.s004.docx]

# **S4 Table. Adjusted Associations between Neighborhood Social Conditions and Mortality, Cox models**

| Hazard ratio (95% CI), P value | | | | | |
| --- | --- | --- | --- | --- | --- |
| Quintiles of social condition measures | Low income ^a^ | Education | Essential workers | Unemployment rate | Crowding housing |
| Quintile 1 (Ref.) | 1.00 | 1.00 | 1.00 | 1.00 | 1.00 |
| Quintile 2 | 1.30 (1.01, 1.68), 0.04 | 1.08 (0.76, 1.54), 0.67 | 1.46 (1.17, 1.81), 0.001 * | 1.07 (0.82, 1.38), 0.63 | 0.63 (0.36, 1.10), 0.11 |
| Quintile 3 | 1.35 (1.04, 1.77), 0.03 * | 1.63 (1.21, 2.20), 0.001 * | 1.40 (1.15, 1.71), 0.001 * | 1.21 (0.94, 1.57), 0.14 | 1.12 (0.71, 1.75), 0.63 |
| Quintile 4 | 1.72 (1.39, 2.14), <0.001 * | 1.82 (1.40, 2.37), <0.001 * | 1.47 (1.24, 1.74), <0.001 * | 1.56 (1.24, 1.97), <0.001 * | 1.22 (0.82, 1.81), 0.33 |
| Quintile 5 | 1.63 (1.31, 2.02), <0.001 * | 1.84 (1.44, 2.36), <0.001 * | 1.42 (1.20, 1.67), <0.001 * | 1.50 (1.19, 1.89), 0.001 * | 1.57 (1.07, 2.30), 0.02 * |
| Age | 1.04 (1.03, 1.04), <0.001 | 1.04 (1.03, 1.04), <0.001 | 1.04 (1.03, 1.04), <0.001 | 1.04 (1.03, 1.04), <0.001 | 1.04 (1.03, 1.04), <0.001 |
| Gender |  |  |  |  |  |
| Female (ref.) | 1.00 | 1.00 | 1.00 | 1.00 | 1.00 |
| Male | 1.19 (1.07, 1.32), 0.001 | 1.19 (1.07, 1.32), 0.001 | 1.19 (1.10, 1.29), 0.001 | 1.20 (1.08, 1.33), 0.001 | 1.19 (1.08 1.32), 0.001 |
| Race |  |  |  |  |  |
| White (ref.) | 1.00 | 1.00 | 1.00 | 1.00 | 1.00 |
| Black | 1.03 (0.89, 1.18), 0.72 | 1.02 (0.89, 1.17), 0.79 | 1.04 (0.90, 1.20), 0.57 | 1.02 (0.88, 1.17), 0.82 | 1.02 (0.89, 1.18), 0.74 |
| Asian | 0.95 (0.76, 1.20), 0.68 | 0.96 (0.76, 1.21), 0.75 | 0.95 (0.75, 1.20), 0.66 | 0.95 (0.76, 1.20), 0.68 | 0.98 (0.78, 1.23), 0.86 |
| Other/unknown | 0.95 (0.83, 1.08), 0.40 | 0.94 (0.83, 1.07), 0.38 | 0.95 (0.84, 1.08), 0.47 | 0.94 (0.83, 1.07), 0.35 | 0.94 (0.83, 1.07), 0.38 |
| Ethnicity |  |  |  |  |  |
| Hispanic (ref.) | 1.00 | 1.00 | 1.00 | 1.00 | 1.00 |
| Non-Hispanic | 0.70 (0.61, 0.80), <0.001 | 0.70 (0.61, 0.80), <0.001 | 0.68 (0.60, 0.79), <0.001 | 0.70 (0.61, 0.80), <0.001 | 0.70 (0.61, 0.81), <0.001 |
| Unknown | 1.23 (1.07, 1.42), 0.003 | 1.23 (1.07, 1.42), 0.003 | 1.23 (1.08, 1.42), 0.004 | 1.24 (1.07, 1.43), 0.003 | 1.24 (1.08, 1.42), 0.003 |
| Comorbidities |  |  |  |  |  |
| Hypertension | 1.20 (1.05, 1.37), 0.007 | 1.19 (1.04, 1.36), 0.01 | 1.19 (1.04, 1.36), 0.009 | 1.19 (1.04, 1.36), 0.01 | 1.19 (1.04, 1.36), 0.01 |
| Diabetes | 1.27 (1.13, 1.42), <0.001 | 1.27 (1.14, 1.42), <0.001 | 1.28 (1.14, 1.43), <0.001 | 1.28 (1.15, 1.44), <0.001 | 1.27 (1.14, 1.43), <0.001 |
| Coronary artery disease | 1.11 (0.98, 1.25), 0.11 | 1.11 (0.98, 1.26), 0.09 | 1.12 (0.99, 1.27), 0.08 | 1.10 (0.97, 1.25), 0.13 | 1.10 (0.97, 1.25), 0.13 |
| Heart failure | 1.39 (1.21, 1.59), <0.001 | 1.39 (1.21, 1.59), <0.001 | 1.39 (1.22, 1.60), <0.001 | 1.40 (1.22, 1.60), <0.001 | 1.39 (1.22, 1.60), <0.001 |
| COPD | 1.08 (0.93, 1.24),  0.31 | 1.07 (0.93, 1.24),  0.34 | 1.08 (0.93, 1.24),  0.30 | 1.07 (0.93, 1.24),  0.36 | 1.07 (0.93, 1.24),  0.34 |
| Asthma | 0.91 (0.77, 1.07), 0.25 | 0.91 (0.77, 1.07), 0.25 | 0.92 (0.78, 1.08), 0.29 | 0.90 (0.76, 1.06), 0.21 | 0.91 (0.77, 1.07), 0.24 |
| Cancer | 1.11 (0.98, 1.26), 0.09 | 1.12 (0.99, 1.26), 0.08 | 1.10 (0.98, 1.25), 0.12 | 1.11 (0.98, 1.25), 0.10 | 1.11 (0.98, 1.25), 0.10 |
| Obesity | 1.28 (1.14, 1.44),  <0.001 | 1.28 (1.14, 1.44),  <0.001 | 1.28 (1.14, 1.44),  <0.001 | 1.28 (1.14, 1.44),  <0.001 | 1.28 (1.14, 1.44),  <0.001 |
| Hyperlipidemia | 0.98 (0.86, 1.10),  0.71 | 0.97 (0.86, 1.10),  0.68 | 0.98 (0.87, 1.11),  0.73 | 0.98 (0.87, 1.12),  0.79 | 0.97 (0.86, 1.10),  0.68 |
| Laboratory tests |  |  |  |  |  |
| Creatinine >1.5 mg/dL | 1.36 (1.22, 1.52), <0.001 | 1.36 (1.22, 1.52), <0.001 | 1.36 (1.22, 1.52), <0.001 | 1.36 (1.21, 1.52), <0.001 | 1.37 (1.22, 1.53), <0.001 |
| White blood cell count < 4×10^3^ cells/μL | 0.76 (0.61, 0.95), 0.02 | 0.76 (0.61, 0.95), 0.01 | 0.76 (0.61, 0.95), 0.01 | 0.75 (0.60, 0.94), 0.01 | 0.75 (0.60, 0.94), 0.01 |
| White blood cell count > 10×10^3^ cells/μL | 1.24 (1.03, 1.50), 0.02 | 1.26 (1.05, 1.52), 0.02 | 1.24 (1.03, 1.49), 0.03 | 1.28 (1.06, 1.54), 0.01 | 1.26 (1.05, 1.53), 0.02 |
| Lymphocyte count < 1×10^3^ cells/μL | 1.09 (0.98, 1.20), 0.11 | 1.09 (0.98, 1.20), 0.12 | 1.08 (0.98, 1.20), 0.13 | 1.09 (0.98, 1.21), 0.10 | 1.09 (0.98, 1.20), 0.11 |
| Platelet count <150 ×10^3^ cells/μL | 1.30 (1.15, 1.46),  <0.001 | 1.30 (1.15, 1.47),  <0.001 | 1.29 (1.15, 1.46),  <0.001 | 1.29 (1.15, 1.46),  <0.001 | 1.30 (1.15, 1.47),  <0.001 |
| Bilirubin ≥ 1.2 mg/dL | 1.33 (1.03, 1.72), 0.03 | 1.33 (1.03, 1.72), 0.03 | 1.31 (1.02, 1.70), 0.04 | 1.34 (1.03, 1.72), 0.03 | 1.33 (1.03, 1.72), 0.03 |
| Aspartate aminotransferase > 40 U/L | 1.54 (1.39, 1.70), <0.001 | 1.52 (1.38, 1.68), <0.001 | 1.53 (1.38, 1.68), <0.001 | 1.55 (1.41, 1.72), <0.001 | 1.53 (1.39, 1.69), <0.001 |
| Albumin < 3.5 g/dl | 1.14 (1.03, 1.27),  <0.001 | 1.16 (1.05, 1.28),  0.005 | 1.16 (1.05, 1.29),  <0.001 | 1.13 (1.02, 1.25),  0.02 | 1.14 (1.03, 1.27),  0.01 |
| Red blood cell distribution width > 13.5% | 0.93 (0.84, 1.03),  0.18 | 0.93 (0.84, 1.03),  0.17 | 0.93 (0.84, 1.03),  0.18 | 0.92 (0.83, 1.03),  0.14 | 0.94 (0.84, 1.04),  0.21 |
| Neutrophil count > 7.4 ×10^3^ cells/μL | 1.27 (1.06, 1.52), 0.01 | 1.26 (1.05, 1.51), 0.01 | 1.28 (1.06, 1.53), 0.009 | 1.24 (1.03, 1.49), 0.02 | 1.25 (1.04, 1.51), 0.02 |

*Notes: SDI: Social Deprivation Index. ^a^ For income, higher quintiles indicate lower income. * indicates FDR q-value < 0.05.*
