# Supplementary material for: Socioeconomic variation in characteristics, outcomes, and healthcare utilization of COVID-19 patients in New York City
Source: PLoS One. 2021 Jul 29;16(7):e0255171. doi: 10.1371/journal.pone.0255171 (PMC8321227; doi:10.1371/journal.pone.0255171)
Supplement: S5 Table — (DOCX) [file pone.0255171.s005.docx]

# **S5 Table. Overall Patient Characteristics by Quintiles of Social Deprivation Index, Long-Term Care Facility Residents**

| Characteristics | Overall  N=4,577 | Social Deprivation Index Quintiles | | | | | P value^a^ |
| --- | --- | --- | --- | --- | --- | --- | --- |
|  |  | Quintile 1  (socially advantaged)  N = 33 | Quintile 2  N = 62 | Quintile 3  N = 81 | Quintile 4  N = 249 | Quintile 5  (socially disadvantaged)  N = 4,152 |  |
| Treatment settings, No. (%) |  |  |  |  |  |  |  |
| Admitted to ambulatory clinics only | 46 (1.0) | 1 (3.0) | 2 (3.2) | 1 (1.2) | 3 (1.2) | 39 (0.9) | 0.22 |
| Admitted to ED only | 99 (2.2) | 4 (12.1) | 5 (8.1) | 4 (4.9) | 5 (2.0) | 81 (2.0) | <0.001* |
| Hospitalized | 4,432 (96.8) | 28 (84.9) | 55 (88.7) | 76 (93.8) | 241 (96.8) | 4,032 (97.1) | 0.003 * |
| Age, median (IQR) | 63  (51-75) | 74  (56-85) | 76  (55-87) | 63  (52-87) | 67  (54-77) | 63  (50-74) | 0.005 * |
| Gender, No. (%) |  |  |  |  |  |  |  |
| Female | 2,188 (47.8) | 17 (51.5) | 29 (46.8) | 41 (50.6) | 113 (45.4) | 1,988 (47.9) | 0.68 |
| Male | 2,388 (52.2) | 16 (48.5) | 33 (53.3) | 40 (49.4) | 136 (54.6) | 2,163 (52.1) | 0.68 |
| Other/Unknown | 1 (0.0) | 0 (0.0) | 0 (0.0) | 0 (0.0) | 0 (0.0) | 1 (0.0) | >0.99 |
| Race, No. (%) |  |  |  |  |  |  |  |
| White | 549 (12.0) | 23 (69.7) | 36 (58.1) | 43 (53.1) | 93 (37.4) | 354 (8.5) | <0.001 * |
| Black | 1,576 (34.4) | 2 (6.1) | 6 (9.7) | 13 (16.1) | 39 (15.7) | 1,516 (36.5) | <0.001 * |
| Asian | 126 (2.8) | 1 (3.0) | 3 (4.8) | 2 (2.5) | 9 (3.6) | 111 (2.7) | 0.59 |
| Other/unknown | 2,326 (50.8) | 7 (21.2) | 17 (27.4) | 23 (28.4) | 108 (43.4) | 2,171 (52.3) | <0.001 * |
| Ethnicity, No. (%) |  |  |  |  |  |  |  |
| Hispanic | 1,718 (37.5) | 1 (3.0) | 4 (6.5) | 10 (12.4) | 67 (26.9) | 1,636 (39.4) | <0.001 * |
| Non-Hispanic | 2,381 (52.0) | 25 (75.8) | 49 (79.0) | 60 (74.1) | 142 (57.0) | 2,105 (50.7) | 0.005 * |
| Unknown | 478 (10.4) | 7 (21.2) | 9 (14.5) | 11 (13.6) | 40 (16.1) | 411 (9.9) | 0.04 |
| BMI, median (IQR) | 28.7  (24.7-33.4) | 24.6  (22.7-27.1) | 26.2  (22.7-30.0) | 26.6  (23.4-30.2) | 28.8  (25.0-33.2) | 28.8  (24.9-33.5) | <0.001 * |
| BMI level, No. (%) |  |  |  |  |  |  |  |
| <18.5 (%) | 152 (3.3) | 4 (12.1) | 2 (3.2) | 7 (8.6) | 3 (1.2) | 136 (3.3) | 0.02 * |
| 18.5-24.9 | 1,020 (22.3) | 15 (45.5) | 25 (40.3) | 26 (32.1) | 57 (22.9) | 897 (21.6) | 0.002 * |
| 25.0-29.9 | 1,414 (30.9) | 8 (24.2) | 19 (30.7) | 23 (28.4) | 76 (30.5) | 1,288 (31.0) | 0.46 |
| >=30.0 | 1,831 (40.0) | 6 (18.2) | 14 (22.6) | 21 (25.9) | 98 (39.4) | 1,692 (40.8) | 0.007 * |
| Missing | 160 (3.5) | 0 (0.0) | 2 (3.2) | 4 (4.9) | 15 (6.0) | 139 (3.4) | 0.63 |
| Comorbidities,  No. (%) |  |  |  |  |  |  |  |
| Hypertension | 3,328 (72.7) | 18 (54.6) | 36 (58.1) | 45 (55.6) | 175 (70.3) | 3,054 (73.6) | 0.01 * |
| Diabetes | 2,289 (50.0) | 9 (27.3) | 21 (33.9) | 20 (24.7) | 106 (42.6) | 2,133 (51.4) | 0.008 * |
| Coronary artery disease | 1,374 (30.0) | 10 (30.3) | 19 (30.7) | 19 (23.5) | 94 (37.8) | 1,232 (29.7) | 0.94 |
| Heart failure | 1,023 (22.4) | 4 (12.1) | 16 (25.8) | 17 (21.0) | 56 (22.5) | 930 (22.4) | 0.16 |
| COPD | 946 (20.7) | 5 (15.2) | 4 (6.5) | 13 (16.1) | 63 (25.3) | 861 (20.7) | 0.43 |
| Asthma | 1,002 (21.9) | 1 (3.0) | 6 (9.7) | 6 (7.4) | 52 (20.9) | 937 (22.6) | 0.007 * |
| Cancer | 1,204 (26.3) | 12 (36.4) | 18 (29.0) | 18 (22.2) | 54 (21.7) | 1,102 (26.5) | 0.20 |
| Obesity | 1,709 (37.3) | 5 (15.2) | 12 (19.4) | 18 (22.2) | 74 (29.7) | 1,600 (38.5) | 0.006 * |
| Hyperlipidemia | 2,362 (51.6) | 17 (51.5) | 33 (53.2) | 36 (44.4) | 136 (54.6) | 2,140 (51.5) | >0.99 |
| Mortality, No. (%) | 1,048 (22.9) | 9 (27.3) | 18 (29.0) | 22 (27.2) | 76 (30.5) | 923 (22.2) | 0.49 |

*Notes: ^a^ P values were calculated by comparing patients from quintile 1 areas and those from quintile 5 areas using χ2 test for categorical variables or Wilcoxon rank-sum test for continuous variables. IQR: interquartile range. * indicates FDR q-value < 0.05*
