# Supplementary material for: Socioeconomic variation in characteristics, outcomes, and healthcare utilization of COVID-19 patients in New York City
Source: PLoS One. 2021 Jul 29;16(7):e0255171. doi: 10.1371/journal.pone.0255171 (PMC8321227; doi:10.1371/journal.pone.0255171)
Supplement: S6 Table — (DOCX) [file pone.0255171.s006.docx]

# **S6 Table. Characteristics and Treatment of Hospitalized Patients by Quintiles of Social Deprivation Index, Long-Term Care Facility Residents**

|  | Overall  N=4,432 | Social Deprivation Index Quintiles | | | | | P value^a^ |
| --- | --- | --- | --- | --- | --- | --- | --- |
|  |  | Quintile 1  (socially advantaged)  N = 28 | Quintile 2  N = 55 | Quintile 3  N = 76 | Quintile 4  N = 241 | Quintile 5  (socially disadvantaged)  N = 4,032 |  |
| Discharge status, No (%) |  |  |  |  |  |  |  |
| Discharged alive | 3,451 (77.9) | 75 (75.0) | 41 (74.6) | 58 (76.3) | 167 (69.6) | 3,164 (78.5) | 0.66 |
| Died in hospital | 981 (22.1) | 7 (25.0) | 14 (25.4) | 18 (23.7) | 73 (30.4) | 869 (21.6) | 0.66 |
| Laboratory Results, median (IQR) and N |  |  |  |  |  |  |  |
| Venous lactate (mmol/L) | 2.1  (1.6-2.9), 3,513 | 1.4  (1.3-1.8), 14 | 2.0  (1.4-2.2), 29 | 1.5  (1.3-2.2), 44 | 2.2  (1.6-3.0), 168 | 2.1  (1.6-2.9), 3,258 | 0.005 * |
| Creatinine (mg/dL) | 1.1  (0.8-1.7), 4,260 | 0.9  (0.7-1.4), 28 | 1.2  (0.8-1.5), 54 | 0.9  (0.7-1.5), 74 | 1.0  (0.8-1.7), 234 | 1.1  (0.8-1.7), 3,870 | 0.27 |
| White blood cell count (×10^3^ cells/μL) | 7.5  (5.5-10.3), 4,260 | 8.2  (5.6-12.6), 28 | 6.9  (4.8-9.9), 54 | 6.9  (5.3-9.8), 74 | 7.9  (5.5-10.7), 234 | 7.4  (5.5-10.3), 3,870 | 0.35 |
| Lymphocyte count (×10^3^ cells/μL) | 1.0  (0.7-1.4), 4,236 | 0.8  (0.5-1.0), 24 | 0.9  (0.6-1.3), 48 | 0.8  (0.7-1.2), 68 | 0.9  (0.7-1.3), 230 | 1.0  (0.7-1.4), 3,866 | 0.009 * |
| Platelet count (×10^3^ cells/μL) | 216  (164-282), 4,260 | 210  (165-301), 28 | 199  (146-236), 54 | 182  (155-232), 74 | 215  (159-280), 234 | 217  (165-284), 3,870 | 0.76 |
| Bilirubin (mg/dL) | 0.3  (0.2-0.5), 4,034 | 0.5  (0.3-0.6), 27 | 0.4  (0.3-0.5), 51 | 0.4  (0.3-0.7), 72 | 0.3  (0.3-0.5), 222 | 0.3  (0.2-0.5), 3,662 | 0.09 |
| Aspartate aminotransferase (U/L) | 40  (27-65), 3,947 | 45  (31-61), 26 | 37  (26- 61), 49 | 40  (29-59), 69 | 42  (29-63), 220 | 40  (27-65), 3,583 | 0.61 |
| Alanine aminotransferase (U/L) | 28  (18-46), 4,047 | 32  (21-47), 27 | 25  (16-45), 51 | 27  (19-42), 72 | 27  (19-46), 222 | 28  (18-46), 3,675 | 0.63 |
| Creatine kinase (U/L) | 161  (83-381), 3,254 | 144  (58-653), 21 | 165  (85-317), 33 | 169  (83-394), 54 | 143  (68-391), 166 | 163  (84-381), 2,980 | 0.74 |
| C-reactive protein (mg/L) | 92  (37-176), 2,745 | 101  (48-145), 20 | 73  (31-149), 37 | 58  (31-133), 54 | 107  (43-180), 1,46 | 93  (37-176), 2,488 | 0.96 |
| Ferritin (ng/mL) | 713  (322-1,420), 2,254 | 734  (288-1,457), 17 | 379  (194-1,122), 35 | 676  (397-1,172), 52 | 732  (339-1,562), 126 | 716  (325-1,424), 2,024 | 0.94 |
| D-dimer (μg/mL) | 1.6  (0.8-3.5), 2,360 | 0.9  (0.5-2.6), 8 | 1.2  (0.6-2.5), 18 | 1.0  (0.6-2.1), 27 | 1.5  (0.8-3.4), 119 | 1.6  (0.8-3.6), 2,188 | 0.49 |
| Cardiac troponin T (ng/L) | 10  (10-20), 3,414 | 10  (10-30), 11 | 10  (10-30), 22 | 10  (10-10), 37 | 10  (10-20), 174 | 10  (10-20), 3,170 | 0.21 |
| Albumin (g/dl) | 3.8  (3.4-4.1), 4,059 | 3.6  (3.6-4.0), 27 | 3.7  (3.5-4.0), 51 | 3.8  (3.5-4.1), 72 | 3.7  (3.3-4.1), 222 | 3.8  (3.4-4.1), 3,687 | 0.08 |
| Red blood cell distribution width (%) | 13.9  (13.0-15.2), 4,258 | 14.3  (13.1-15.4), 28 | 13.9  (12.9-14.6), 54 | 14.0  (13.2-15.3), 74 | 13.7  (12.8-14.9), 234 | 13.9  (13.0-15.2), 3,868 | 0.75 |
| Neutrophil count (×10^3^ cells/μL) | 5.6  (3.9-8.3), 4,236 | 6.3  (3.9-11.1), 24 | 4.7  (3.3-8.2), 48 | 5.4  (3.7-7.9), 68 | 6.0  (4.1-8.6), 230 | 5.6  (3.9-8.3), 3,866 | 0.38 |
| Treatment and procedures | |  |  |  |  |  |  |
| Length of stay, median (IQR) | 7  (4-11) | 8  (6-11) | 7  (4-14) | 7  (4-12) | 7  (4-11) | 7  (4-11) | 0.077 |
| ICU care (%) | 39 (0.9) | 1 (3.6) | 2 (3.6) | 7 (9.2) | 11 (4.6) | 18 (0.5) | 0.12 |
| Invasive mechanical ventilation (%) | 768 (17.0) | 7 (25.0) | 7 (12.7) | 12 (15.8) | 55 (22.9) | 687 (17.0) | 0.31 |
| Respiratory Ventilation, Less than 24 Consecutive Hours (%) | 139 (3.1) | 0 (0.0) | 4 (4.3) | 2 (2.6) | 9 (3.8) | 124 (3.1) | >0.99 |
| Respiratory Ventilation, 24-96 Consecutive Hours (%) | 164 (3.7) | 1 (3.6) | 0 (0.0) | 3 (4.0) | 11 (4.6) | 149 (3.7) | >0.99 |
| Respiratory Ventilation, Greater than 96 Consecutive Hours (%) | 463 (10.5) | 5 (17.9) | 3 (5.5) | 7 (9.2) | 31 (12.9) | 417 (10.3) | 0.21 |
| Renal replacement therapy (%) | 379 (8.6) | 2 (7.1) | 3 (5.5) | 4 (5.3) | 19 (7.9) | 351 (8.7) | >0.99 |
| Vasopressor use (%) | 205 (4.6) | 0 (0.0) | 1 (1.8) | 8 (10.5) | 17 (7.1) | 179 (4.4) | 0.63 |
| Hydroxychloroquine (%) | 2,933 (66.2) | 8 (28.6) | 18 (32.7) | 32 (42.1) | 145 (60.4) | 2,730 (67.7) | <0.001 * |
| Steroid (%) | 1,243 (28.1) | 5 (17.9) | 15 (27.3) | 13 (17.1) | 66 (27.5) | 1,144 (28.4) | 0.29 |
| Discharge destinations (%) |  |  |  |  |  |  |  |
| Home | 2,214 (64.2) | 8 (38.1) | 20 (48.8) | 31 (53.5) | 88 (52.7) | 2,067 (65.3) | 0.009 * |
| Hospice | 37 (1.1) | 4 (19.1) | 5 (12.2) | 4 (6.9) | 2 (1.2) | 22 (0.7) | <0.001 * |
| Other acute inpatient hospital | 24 (0.7) | 0 (0.0) | 0 (0.0) | 0 (0.0) | 3 (1.8) | 21 (0.7) | >0.99 |
| Long-term care facilities/rehab | 1,095 (31.7) | 9 (42.9) | 16 (39.0) | 21 (36.2) | 68 (40.7) | 981 (31.0) | 0.24 |
| Other | 81 (2.4) | 0 (0.0) | 0 (0.0) | 2 (3.5) | 6 (3.6) | 73 (2.3) | >0.99 |

*Notes: ^a^ P values were calculated by comparing patients from quintile 1 areas and those from quintile 5 areas using χ2 test for categorical variables or Wilcoxon rank-sum test for continuous variables. IQR: interquartile range. * indicates FDR q-value < 0.05.*
