# Supplementary material for: Socioeconomic variation in characteristics, outcomes, and healthcare utilization of COVID-19 patients in New York City
Source: PLoS One. 2021 Jul 29;16(7):e0255171. doi: 10.1371/journal.pone.0255171 (PMC8321227; doi:10.1371/journal.pone.0255171)
Supplement: S7 Table — (DOCX) [file pone.0255171.s007.docx]

# **S7 Table. Healthcare Utilization 30-Day after Discharge by Quintiles of Social Deprivation Index, Long-Term Care Facility Residents**

|  |  | Social Deprivation Index Quintiles | | | | |  |
| --- | --- | --- | --- | --- | --- | --- | --- |
| Hospitalized patients | Overall  N=3,454 | Quintile 1  N = 21 | Quintile 2  N = 41 | Quintile 3  N = 58 | Quintile 4  N = 167 | Quintile 5  N = 3,167 | P value ^a^ |
| Any ambulatory visits (%) | 38.3 | 33.3 | 22.0 | 20.7 | 26.4 | 39.4 | 0.66 |
| Any emergency department visits (%) | 5.9 | 0.0 | 7.3 | 1.7 | 4.8 | 6.1 | 0.63 |
| Any hospitalizations (%) | 9.0 | 4.8 | 12.2 | 8.6 | 4.8 | 9.2 | 0.72 |
| Patients presented to ED without hospitalization | Overall  N= 99 | Quintile 1  N = 4 | Quintile 2  N = 4 | Quintile 3  N = 4 | Quintile 4  N = 3 | Quintile 5  N = 84 | P value ^a^ |
| Any ambulatory visits (%) | 28.0 | 0.0 | 0.0 | 25.0 | 0.0 | 32.1 | 0.31 |
| Any emergency department visits (%) | 11.0 | 0.0 | 0.0 | 25.0 | 0.0 | 11.9 | >0.99 |
| Any hospitalizations (%) | 8.0 | 0.0 | 0.0 | 0.0 | 0.0 | 9.5 | >0.99 |
| Patients presented to ambulatory clinics only | Overall  N= 46 | Quintile 1  N = 1 | Quintile 2  N = 2 | Quintile 3  N = 1 | Quintile 4  N = 2 | Quintile 5  N = 40 | P value ^a^ |
| Any ambulatory visits (%) | 66.0 | 0.0 | 0.0 | 0.0 | 50.0 | 73.2 | 0.29 |
| Any emergency department visits (%) | 0.0 | 0.0 | 0.0 | 0.0 | 0.0 | 0.0 | - |
| Any hospitalizations (%) | 10.6 | 0.0 | 0.0 | 0.0 | 0.0 | 12.2 | >0.99 |

*Notes: ^a^ P values were calculated by comparing patients from quintile 1 areas (socially advantaged) and those from quintile 5 areas (socially disadvantaged) using χ2 test for categorical variables or Wilcoxon rank-sum test for continuous variables.*
